# Induction of ROS-mediated genomic instability, mitochondrial deploarization and *p53*-independent mitochondrial apoptotic cell death by bioactive glass nanoparticles in human A431 epidermoid skin cancer cells

**DOI:** 10.1186/s12885-026-15866-x

**Published:** 2026-04-02

**Authors:** Hanan R H Mohamed, Shahd Mosaad, Aya A. Osman, Alaa H. Elsewedy, Habiba M. Zaki, Mayada E. Borai, Gehan Safwat

**Affiliations:** 1 Department of Zoology, Faculty of Science, Cairo University, Giza, Egypt; 2 Faculty of Biotechnology, October University for Modern Sciences and Arts (MSA), 6th of October City, Egypt

**Keywords:** Bioactive glass nanoparticles, Epidermoid skin cancer, A431 cells, MTT assay, Oxidative stress, Genomic instability, Mitochondrial dysfunction and apoptosis induction

## Abstract

Epidermoid skin cancer remains a significant clinical challenge due to the limited selectivity, systemic toxicity, and resistance associated with conventional chemotherapies. Bioactive glass nanoparticles (BGNPs), widely recognized for their regenerative capacity and excellent biocompatibility, have recently gained attention in nanomedicine. However, their anticancer potential, particularly in epidermoid skin cancer, has not yet been investigated. Therefore, the present study was conducted to systematically evaluate, for the first time, the cytotoxic effects and underlying molecular mechanisms of BGNPs in human A431 epidermoid carcinoma cells.

Cancerous A431cells were treated with BGNPs across a concentration range of 7.8–1000 µg/ml, and cytotoxicity was quantified using the MTT assay, revealing a potent concentration-dependent reduction in cell viability with an IC50 value of 187.81 µg/ml. Mechanistic analyses demonstrated that A431 cell exposure to BGNPs at the IC50 concentration led to a significant increase in intracellular reactive oxygen species (ROS), as detected using the 2′,7′-dichlorodihydrofluorescein diacetate (DCFH-DA) assay, accompanied by severe mitochondrial membrane depolarization and dramatic genomic DNA damage, as confirmed by Rhodamine-123 staining and alkaline comet assay. Apoptosis was validated by DAPI staining and chromatin diffusion assays, which demonstrated characteristic nuclear condensation and fragmentation, along with significant increases in the proportion of apoptotic A431 cells following BGNPs treatment compared to untreated control cells. Furthermore, qRT-PCR analysis showed significant downregulation of apoptotic *p53* alongside marked upregulation of anti-apoptotic *Bcl-2* and mitochondrial *ND3* genes, indicating disruption of mitochondrial and apoptotic regulatory pathways. Conclusion: Collectively, this study provides novel mechanistic evidence that BGNPs induce potent cytotoxicity in A431 cells through a ROS-mediated, mitochondria-dependent apoptotic pathway. Despite being limited to a single in vitro cell line, these findings highlight BGNPs as promising multifunctional anticancer candidates, warranting further in vitro studies across additional skin cancer models and normal keratinocyte cell lines alongside *n vivo* validation and exploration in combination therapeutic strategies.

## Introduction

Epidermoid skin cancer, clinically referred to as cutaneous squamous cell carcinoma (cSCC), is the second most common type of non-melanoma skin cancer, accounting for approximately 20% of all cutaneous malignancies [1, 2]. The global incidence of cSCC has been steadily rising, primarily due to to increased life expectancy, cumulative exposure to ultraviolet (UV) radiation, the widespread use of immunosuppressive therapies, and exposure to environmental carcinogens, particularly among fair-skinned individuals and immunocompromised populations [2, 3]. Although many early-stage cases are effectively managed through surgical excision, cSCC continues to impose a substantial clinical burden due to its high recurrence rates, potential for local tissue invasion, and risk of reginal and distant metastasis, particularly in high-risk or advanced-stage tumors [4].

Despite the availability of conventional treatment modalities, including surgical excision, radiotherapy, and chemotherapy, therapeutic outcomes remain unsatisfactory in many cases, particularly for advanced or recurrent lesions. Chemotherapy, in particular, is limited by several well-established drawbacks, including poor tumor selectivity, systemic toxicity, drug resistance development, and low efficacy in long-term disease control [3–6]. These challenges are further compounded by adverse effects such as nephrotoxicity, myelosuppression, and peripheral neuropathy, which not only compromise treatment efficacy but also significantly diminish patient quality of life and reduce adherence to therapy [4]. Given these persistent limitations, there is a critical and urgent need to develop safer, more targeted, and mechanistically distinct therapeutic strategies capable of selectively eradicating malignant cells while sparing healthy skin tissue. Advancing such approaches is essential for improving both clinical outcomes and the overall well-being of patients affected by this increasingly prevalent malignancy.

In this context, nanotechnology-based therapeutic approaches have gained increasing attention for their potential to improve therapeutic precision, enhance cellular uptake, and reduce systemic toxicity in cancer treatment [7, 8]. Among emerging nanomaterials, bioactive glass nanoparticles (BGNPs) stand out due to their excellent biocompatibility, biodegradability, and ion-releasing capabilities that allow them to interact with cells in a dynamic and tunable manner [9–11]. Upon exposure to physiological fluids, BGNPs release biologically active ions such as calcium, silicon, sodium and phosphate, which can modulate a variety of cellular processes, including oxidative stress induction, mitochondrial membrane disruption, and activation of cell death signaling pathways [11–13].

Although the regenerative applications of BGNPs, particularly in bone repair, tissue engineering, and orthopedic implants, are well established [9, 14, 15], recent studies have begun to reveal their potential anticancer properties. Emerging evidence indicates that BGNPs can exert direct cytotoxic effects on cancer cells through non-drug-mediated mechanisms, including the generation of reactive oxygen species (ROS), induction of genomic instability, and activation of apoptotic pathways [16–18]. Notably, BGNPs have demonstrated selective cytotoxicity against human Hep-G2 hepatocellular carcinoma and A549 non-small cell lung cancer cells, while showing minimal toxicity toward normal human skin fibroblasts and melanocytes (HFB4) [16, 17]. Despite these encouraging findings, the therapeutic potential of BGNPs in cutaneous carcinomas, particularly epidermoid skin cancer, remains unexplored. This lack of data highlights a critical gap in nanomedicine research and underscores the need for systematic investigations into the mechanisms of action, cellular selectivity, and biosafety of BGNPs in epithelial malignancies.

Despite the growing interest in nanotechnology-based cancer therapies, there remains a significant lack of systematic mechanistic studies evaluating BGNPs in relevant cancer models. Notably, no studies to date have investigated the effects of BGNPs on epidermoid carcinoma cells, leaving it unclear whether their reported selective cytotoxicity in bone-related tumors can be extended to epithelial-derived cancers. Furthermore, existing studies are often limited by methodological variability, including differences in nanoparticle synthesis, size distribution, surface properties, and experimental protocols, which hinder reproducibility and cross-study comparisons. Importantly, due to the distinct physicochemical properties of nanoparticles, biological responses observed in bulk systems cannot be directly extrapolated to the nanoscale [10, 11]. Collectively, these limitations underscore the urgent need for standardized, cancer-specific investigations to evaluate the cytotoxic, genotoxic, and apoptotic effects of BGNPs, particularly in epidermoid skin cancer models. Such research is essential to determine whether BGNPs can be harnessed as novel, selective nanotherapeutics for the treatment of epithelial malignancies.

To address these gaps, the present study provides the first comprehensive evaluation of the cytotoxic and mechanistic effects of BGNPs in human A431 epidermoid carcinoma cells. In addition to assessing cytotoxicity, this work systematically investigates key mechanistic endpoints, including intracellular ROS generation, mitochondrial membrane integrity, genomic stability, and apoptosis induction, using a well-integrated panel of biochemical and molecular assays. Importantly, unlike previous studies that have primarily focused on the regenerative or drug-delivery applications of BGNPs, this study uniquely examines their role as standalone anticancer agents, independent of any chemotherapeutic loading. By elucidating the ROS–mitochondria–DNA damage axis as a central mechanism underlying BGNP-induced cytotoxicity, this study provides novel mechanistic insights into their mode of action in epidermoid skin cancer. These findings not only expand the current understanding of BGNPs beyond their conventional applications but also establish a previously unexplored therapeutic potential in epithelial malignancies, supporting their further development as selective and multifunctional nanotherapeutics for skin cancer treatment.

## Materials and methods

### Chemicals

BGNPs in fine powder form were obtained from Nanotech Company (6th October City, Cairo, Egypt). For use, BGNPs were accurately weighed, suspended in dimethyl sulfoxide (DMSO; Sigma-Aldrich, USA), and ultrasonicated for 15–20 min to ensure uniform dispersion. Key reagents, including DMSO, 3-(4,5-dimethylthiazol-2-yl)-2,5-diphenyl tetrazolium bromide (MTT), and trypan blue dye, were sourced from Sigma-Aldrich (St. Louis, MO, USA). Cell culture media and supplements, such as Dulbecco’s Modified Eagle Medium (DMEM), HEPES buffer, L-glutamine, gentamycin, and Trypsin-EDTA, were purchased from Lonza (Belgium) and supplemented with 10% fetal bovine serum (FBS) and 1% gentamycin. Phenol red-free media were used to avoid spectrophotometric interference. All reagents were freshly prepared, and procedures were conducted under aseptic conditions in a Class II biosafety cabinet to maintain sterility and reproducibility.

### Characterization of the used BGNPs

The used BGNPs in this were characterized using multiple physicochemical techniques to assess their structure, size, surface charge, and morphology. X-ray diffraction (XRD) analysis was performed using an XPERT-PRO diffractometer (PANalytical, Netherlands) with Cu Kα radiation to determine the crystalline or amorphous nature of the particles by analyzing characteristic diffraction peaks. Dynamic light scattering (DLS) using a Malvern Zetasizer Nano Series (Malvern Instruments, USA) was employed to measure the hydrodynamic size distribution and zeta potential, providing insights into particle stability and surface charge BGNPs suspension, key factors influencing nanoparticle-cell interactions. Transmission electron microscopy (TEM) was carried out with a Tecnai G20 Super Twin microscope (FEI, USA) at 200 kV to visualize particle morphology and confirm nanoscale dimensions. Samples were prepared by air-drying a drop of BGNP suspension on carbon-coated copper grids. TEM imaging revealed particle shape, size, and aggregation state, complementing DLS data and confirming nanoparticle characteristics.

### Cultivation of A431 epidermoid skin cancer cells

Human A431 epidermoid skin cancer cells were procured from the Regional Center for Mycology and Biotechnology, Al-Azhar University (Cairo, Egypt). Cancerous A431 cells were cultured separately in high-glucose DMEM (4.5 g/L), supplemented with 10% heat-inactivated FBS, 50 µg/mL gentamycin, 100 U/mL penicillin, and 100 µg/mL streptomycin to ensure optimal growth and sterility. Cultured cells were maintained at 37 °C in a humidified incubator with 5% CO₂. Media were refreshed every 2–3 days, and subculturing was performed 2–3 times weekly using 0.25% trypsin-EDTA upon reaching 70–80% confluence. Only healthy, exponentially growing cells with viability ≥ 90% were used for all experimental procedures.

### Cytotoxicity analysis using MTT assay

The cytotoxic effect of BGNPs on human epidermoid A431 carcinoma cells was assessed using the MTT assay following the protocols of Mosmann [19], , El-Zahabi et al. [20], and Abdelsalam et al. [21]. A431 cells were seeded into sterile 96-well flat-bottom plates (Falcon, NJ, USA) at a density of 1 × 10^4^ cells per well in 100 µL of complete DMEM medium containing 10% heat-inactivated FBS, 1% L-glutamine, 100 U/mL penicillin, and 100 µg/mL streptomycin. After 24 h of incubation at 37 °C in a humidified 5% CO_2_ atmosphere to allow cell adhesion, the medium was replaced with fresh DMEM containing serial two-fold dilutions of BGNPs (7.8, 15.6, 31.25, 62.5, 125, 250, 500, and 1000 µg/mL). Each concentration was tested in triplicate. Control wells received medium without BGNPs. After 72 h of exposure, the medium was aspirated and replaced with 100 µL phenol red-free DMEM. Then, 10 µL of 12 mM MTT stock solution (5 mg/mL in PBS) was added to each well. Plates were incubated for 4 hours at 37 °C in the dark, allowing viable cells to reduce MTT to insoluble purple formazan. Following incubation, 85 µL of the medium was gently removed, and 50 µL of DMSO was added to solubilize the formazan crystals. The plates were incubated for 10 min at 37 °C, and absorbance was read at 590 nm using a microplate reader (SunRise, TECAN, USA). Cell viability (%) was calculated using the formula:

$$\mathrm{Cell}\;\mathrm{viability}\;(\%)\;=\;(\mathrm{ODt}\;/\mathrm{ODc})\;\times\;100$$

where ODt is the mean absorbance of treated wells and ODc is that of control wells, and the IC50 value was determined using non-linear regression analysis (GraphPad Prism, San Diego, CA, USA) from three independent experiments.

### Treatment and preparation of A431 cancerous cells for molecular analysis

Human epidermoid A431 skin cancer cells were seeded in T25 flasks using DMEM supplemented with 1% L-glutamine, 10% heat-inactivated FBS, and antibiotics: 100 µg/mL streptomycin and 100 U/mL penicillin. Cancerous A431 cells were maintained at 37 °C in a humidified 5% CO_2_ atmosphere until reaching 70–80% confluence. Cultures were then divided into: untreated control cells that were treated with < 0.1% DMSO, and BGNPs-treated A431 cells that were exposed to the IC50 concentration of tdetermined by MTT assay. Both untreated and BGNPs-treated cells were incubated for 72 h under the same conditions. After treatment, cells were detached using 0.25% trypsin-EDTA and collected by centrifugation at 1,500 rpm for 5 min at 4 °C. Pellets were washed twice with ice-cold PBS (pH 7.4) to eliminate residual media and BGNPs, then resuspended in phosphate buffered saline (PBS) and stored at − 80 °C for subsequent molecular and biochemical assays. All treatments were performed in triplicate to ensure reproducibility and statistical reliability.

### Estimation of genomic DNA damage induction in A431 cells

Induction of genomic DNA damage in A431 epidermoid skin cancer cells following treatment with BGNPs at the IC50 concentration for 72 h was quantitatively assessed using the alkaline single-cell comet assay based on the standardized protocols described by Tice et al. [22] and Langie et al. [23]. Briefly, 15 µL of A431 cell suspension containing approximately 10,000 cells was mixed with 60 µL of 0.5% low-melting-point agarose (prepared in PBS at 37 °C) and immediately spread onto microscope slides pre-coated with 1% normal-melting-point agarose. The slides were left to solidify at room temperature for 30 min. Subsequently, they were immersed in cold lysis buffer (2.5 M NaCl, 100 mM EDTA, 10 mM Tris-HCl, pH 10) containing 1% Triton X-100 and 10% DMSO, and lysed for 24 h at 4 °C in the dark to prevent additional DNA damage. Slides were then incubated in alkaline electrophoresis buffer (300 mM NaOH, 1 mM EDTA, pH > 12) for 15 min to unwind DNA, followed by electrophoresis at 25 V and 300 mA for 30 min at 4 °C. After electrophoresis, slides were neutralized with 0.4 M Tris-HCl buffer (pH 7.5) for 5 min, fixed in cold ethanol for 5 min, and air-dried. Slides were stained with 50 µL of ethidium bromide (20 µg/mL) and analyzed under fluorescence microscopy. Fifty randomly selected nuclei per sample were imaged and analyzed using COMETSCORE™ software. DNA damage was quantified through three parameters: tail length (extent of DNA migration), % DNA in tail (percentage of DNA in the comet tail), and tail moment (integrated measure of tail length and DNA content). Results are presented as mean ± SD from three independent experiments, with statistical analysis performed to evaluate the genotoxic effects of BGNPs treatment on A431 cells.

### Estimation of intracellular ROS generation in A431 skin cancer cells

The intracellular ROS generation in A431 epidermoid skin cancer cells following 72-hour exposure to the IC50 concentration of BGNPs was quantitatively measured using the cell-permeable fluorescent probe 2,7-dichlorofluorescin diacetate (2,7-DCFH-DA), according to the protocol described by Siddiqui et al. [24]. For ROS detection, equal volumes of the A431 cell suspension and 20 µM 2,7-DCFH-DA solution were gently mixed in sterile microcentrifuge tubes and incubated in the dark at room temperature for 30 min. During this incubation, the non-fluorescent probe freely entered the cells and was enzymatically deacetylated by intracellular esterases to form non-fluorescent 2,7-dichlorofluorescin (DCFH). In the presence of ROS, DCFH was oxidized to the fluorescent compound 2,7-dichlorofluorescein (DCF). The intensity of the green fluorescence emitted by DCF served as a direct indicator of intracellular ROS levels. Following incubation, the stained cells were gently spread onto clean, pre-labeled glass microscope slides to form a thin monolayer and examined under an epifluorescence microscope equipped with the appropriate filters for DCF detection. Fluorescent images were captured at 200× magnification from randomly selected fields, using consistent exposure settings across all samples. The fluorescence intensity, reflecting intracellular ROS production, was analyzed using Fiji (ImageJ) software. Relative fluorescence intensities were compared between BGNPs-treated and untreated control cells to quantify ROS generation. All experiments were performed in triplicate to ensure statistical validity and reproducibility of the data.

### Estimation of mitochondrial deplorization in A431 skin cancer cells

Mitochondrial membrane potential, an essential indicator of mitochondrial integrity and early apoptotic events, was quantitatively assessed in A431 epidermoid skin cancer cells 72-hour following treatment with BGNPs at the IC50 concentration. The analysis was performed using Rhodamine-123, a cationic, cell-permeable fluorescent dye that selectively accumulates in polarized mitochondria, following the protocol of Zhang et al. [25] as follow: Equal volumes of the cell suspension and Rhodamine-123 working solution (10 µg/mL) were gently mixed in sterile, light-protected microcentrifuge tubes and incubated at 37 °C for 1 h in the dark to ensure dye uptake by active mitochondria. Following incubation, the cells were washed twice with cold PBS to remove excess dye and reduce background fluorescence. An aliquot of the stained suspension was mounted onto pre-cleaned, labeled glass slides, spread into a thin monolayer, and immediately covered with sterile coverslips. The slides were examined under an epifluorescence microscope equipped with appropriate filters for Rhodamine-123 detection. Fluorescent images were captured at 200× magnification from randomly selected fields using consistent exposure settings across all samples. The fluorescence intensity of Rhodamine-123, reflecting the degree of mitochondrial polarization, was quantified using Fiji (ImageJ) software. A marked decrease in fluorescence in BGNPs-treated cells, compared to untreated control cells, indicated mitochondrial depolarization, a hallmark of early mitochondrial dysfunction and apoptosis. All experiments were conducted in triplicate, and results were expressed as mean ± SD to ensure reproducibility and statistical significance.

### Detection of apoptosis induction in A431 skin cancer cells

Apoptosis, or programmed cell death, is a fundamental cellular process that plays a critical role in maintaining tissue homeostasis and eliminating damaged or cancerous cells. The induction of apoptosis is a key therapeutic goal in cancer treatment, and its detection provides valuable insights into the cytotoxic effects of anticancer agents. In this study, apoptosis induction in A431 epidermoid skin cancer cells following treatment with BGNPs at the IC50 concentration was evaluated using two complementary methods: the chromatin diffusion assay, which detects DNA fragmentation associated with apoptotic signaling, and 4′,6-diamidino-2-phenylindole (DAPI) nuclear staining, which identifies morphological hallmarks of apoptosis such as chromatin condensation and nuclear fragmentation.

### Chromatin diffusion assay

The chromatin diffusion assay is based on the principle that apoptotic cells contain alkali-labile DNA sites, which, under alkaline conditions, lead to DNA fragmentation and diffusion within an agarose matrix. This diffusion produces a characteristic halo surrounding the nucleus, distinguishing apoptotic cells from intact ones [26]. Microscope slides were pre-coated with a thin layer of 0.7% normal-melting-point agarose. A431 cells were mixed with low-melting-point agarose and carefully layered onto the pre-coated slides to embed the cells. After air drying, the slides were immersed in cold lysis buffer for 10 min to remove cellular contents, preserving only the nuclear DNA. Slides were then neutralized with Tris buffer, fixed in cold ethanol, and stained with ethidium bromide, a fluorescent DNA dye. Under a fluorescence microscope, cells exhibiting diffuse halos were identified as apoptotic. A total of 1000 cells per sample were analyzed, and the percentage of apoptotic cells was calculated. All experiments were conducted in triplicate.

### DAPI nuclear staining

To further confirm apoptosis, nuclear morphology was examined using DAPI, a fluorescent dye that binds strongly to DNA and reveals changes such as chromatin condensation and nuclear fragmentation [27]. A431 cells were seeded in 96-well plates (1 × 10^4^ cells/well) and treated with BGNPs at the IC50 concentration for 72 h. Following treatment, cells were washed with PBS and fixed with 4% paraformaldehyde. After fixation, cells were stained with DAPI (1 µg/mL in PBS) for 1 h in the dark, washed, and immediately examined under a fluorescence microscope at 200× magnification from randomly selected fields with a DAPI filter. Apoptotic cells were identified by bright, condensed chromatin, fragmented nuclei, and apoptotic body formation, while non-apoptotic cells displayed diffuse and uniform nuclear staining. The percentage of apoptotic cells was calculated by counting 1000 cells per group. All experiments were performed in triplicate, and data were reported as mean ± SD.

### Expression analysis of p53, ND3 and Bcl2 genes in A431 skin cancer cells

The impact of BGNPs on mitochondrial function and apoptosis induction in A431 skin cancer cells was also assessed by measuring the mRNA expression level of pro-apoptotic *p53* gene, mitochondrial *ND3* gene (involved in electron transport), and anti-apoptotic *Bcl2* gene after 72 h of A431 cell treatment with the IC50 concentration of BGNPs using quantitative real-time PCR (qRT-PCR). For gene expression analysis, total RNA was extracted from both BGNPs-treated and untreated (control) A431 cells using the GeneJET RNA Purification Kit (Thermo Fisher Scientific, USA), following the manufacturer’s instructions. The purity and concentration of the extracted RNA were determined using a NanoDrop spectrophotometer. From each sample, 1 µg of total RNA was then reverse transcribed into cDNA using the High-Capacity cDNA Reverse Transcription Kit (Applied Biosystems, USA). Finally, qRT-PCR reactions were performed using SYBR Green PCR Master Mix and gene-specific primers listed in Table 1 [28–30], on a StepOnePlus Real-Time PCR System (Applied Biosystems), under optimized cycling conditions. GAPDH was used as the internal reference gene for normalization. Relative expression levels of *p53*,* ND3*, and *Bcl2* were calculated using the comparative Ct (ΔΔCt) method. Each reaction was run in triplicate, and results were expressed as mean ± SD from three independent experiments. This analysis provided valuable insights into how BGNPs modulate apoptotic signaling and mitochondrial gene expression in A431 skin cancer cells.

**Table 1:** Sequences of primers used in qRT-PCR

Gene	Strand	Primer’s sequences
GAPDH	Forward	5’-GAAGGTGAAGGTCGGAGTCA-3’
	Reverse	5’-GAAGATGGTGATGGGATTTC-3’
ND3	Forward	5’-CGCCGCCTGATACTGGCAT-3’
	Reverse	5’-CTAGTATTCCTAGAAGTGAG-3’
BCL-2	Forward	5’-TCCGATCAGGAAGGCTAGAGT-3’
	Reverse	5’-TCGGTCTCCTAAAAGCAGGC-3’
P53	Forward	5’-CAGCCAAGTCTGTGACTTGCACGTAC-3’
	Reverse	5’-CTATGTCGAAAAGTGTTTCTGTCATC-3’

### Statistical analysis

All results in the current study are presented as mean ± SD and were statistically analyzed using the Statistical Package for the Social Sciences (SPSS) software. An unpaired two-tailed *Student’s t-test* was conducted to assess the statistical differences in DNA damage level, gene expression levels, apoptotic cell counts, and other measured parameters between BGNPs-treated and untreated A431 skin cancer cells at the probability level of 0.05 (*p* < 0.05).

## Results

### Characterization of the tested BGNPs

**Fig. 1:**
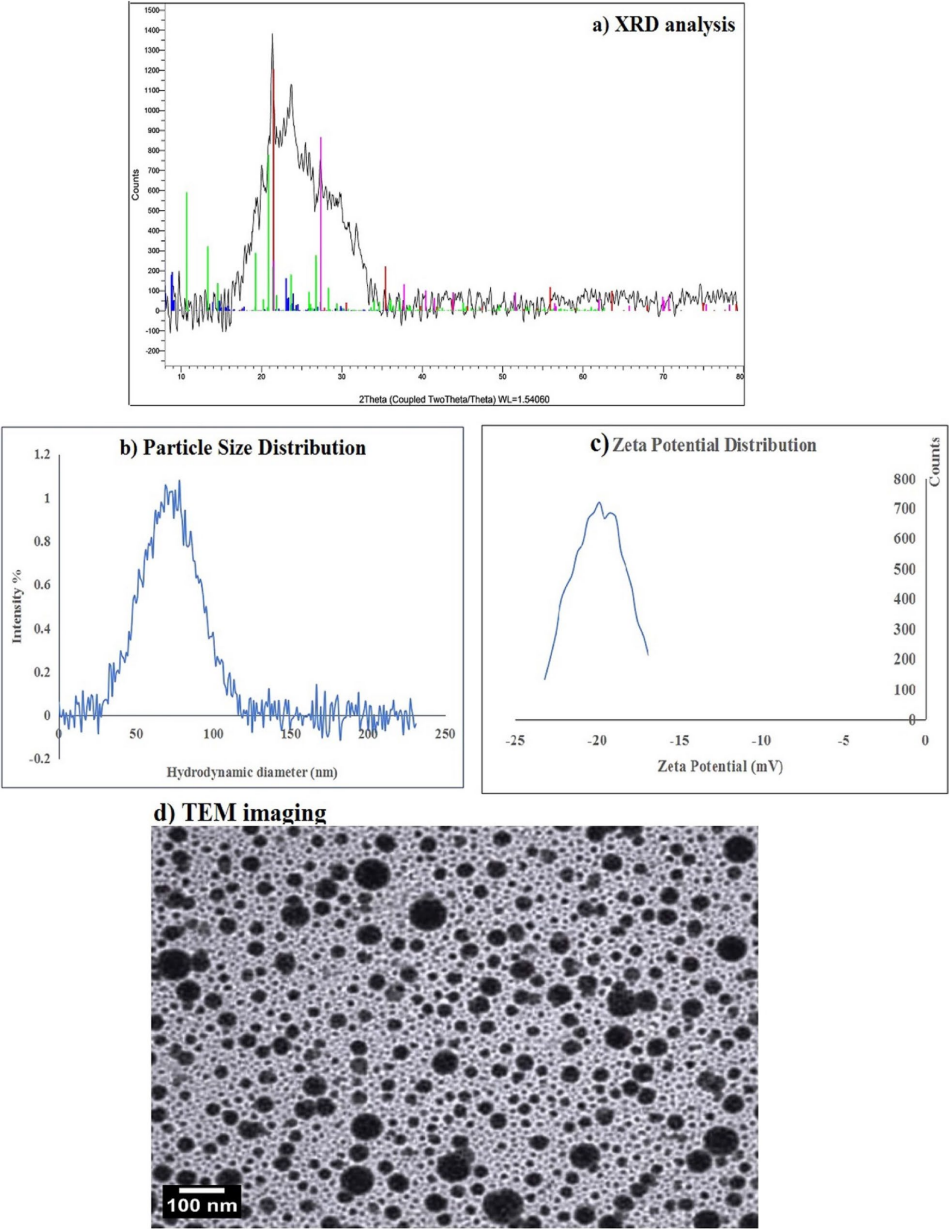
Characterization of BGNPs using (**a**) XRD analysis, (**b**) particle size distribution, (**c**) Zeta potential distribution and (**d**) TEM imaging

The diffraction profile of BGNPs obtained through X-ray diffraction (XRD) analysis confirmed their amorphous glassy nature. As displayed in Fig. 1, a broad diffuse halo was observed across the diffraction angle (2θ) range of 15.48° to 35.11°, with characteristic peaks appearing at 15.48°, 17.22°, 21.63°, 24.06°, 27.84°, 30.55°, and 35.11°. This diffraction pattern is characteristics of a non-crystalline, amorphous structure, characterized by the absence of long-range order. Such a structure is typical of bioactive glasses and is often linked to improved biological performance due to the higher surface area and reactivity. DLS analysis revealed that BGNPs had a particle size range of approximately 35.33–105.99 nm, with an average hydrodynamic diameter of 73.3 nm (Fig. 1). The polydispersity index (PDI) was measured at 0.27, indicating a uniform and narrow particle size distribution. Zeta potential measurements revealed that the BGNPs possessed a negative surface charge of − 19.89 mV, reflecting moderate suspension stability and confirming the inherently anionic nature of BGNPs (Fig. 1). Moreover, morphological examination using TEM analysis confirmed that the BGNPs were predominantly spherical, well-dispersed, and had smooth surface morphology with minimal agglomeration, reflecting efficient synthesis and stabilization. The average particle size measured by TEM was approximately 31.5 nm, verifying the nanoscale nature of the particles (Fig. 1). These physicochemical features are directly relevant to BGNPs biological activity, as the small size and high surface area facilitate cellular uptake, while the amorphous structure and ion-release capability promote ROS generation, mitochondrial disruption, and apoptosis induction in cancer cells.

### BGNPs cause pronounced concentration dependent cytotoxicity in A431 cancer cells

**Fig. 2:**
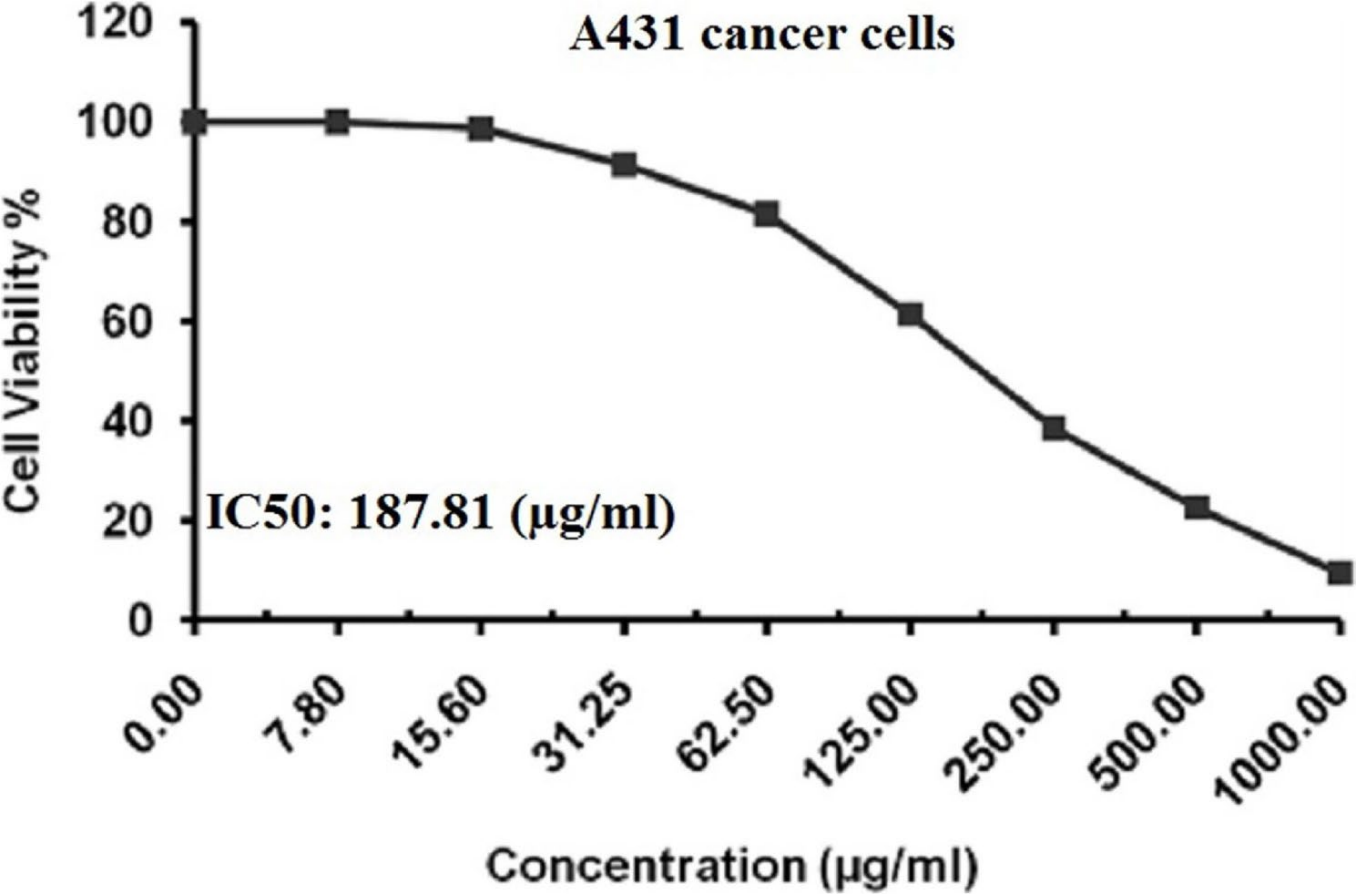
Cytotoxicity of BGNPs on human A431 epidermoid carcinoma cells measured by the MTT assay. Cells were exposed for 72 h to two-fold increasing concentrations of BGNPs (7.8, 15.6, 31.25, 62.5, 125, 250, 500, and 1000 µg/ml). Data are presented as mean ± SD of three independent experiments (*n* = 3)

MTT assay analysis revealed a notable, concentration-dependent reduction in the viability of A431 epidermoid skin cancer cells after 72 h of exposure to increasing two-fold concentrations of BGNPs (7.8, 15.6, 31.25, 62.5, 125, 250, 500, and 1000 µg/mL), as illustrated in Fig. 2. The IC50 value for A431 cells was determined to be 187.81 µg/ml, confirming the cytotoxic efficacy of BGNPs against this cancer cell type. These findings support the therapeutic promise of BGNPs as a potent anticancer agent against epidermoid carcinoma and warrant further mechanistic investigations at the IC50 concentration to explore their underlying mode of action.

### BGNPs cause severe genomic DNA damage in A431 cancer cells

**Fig. 3:**
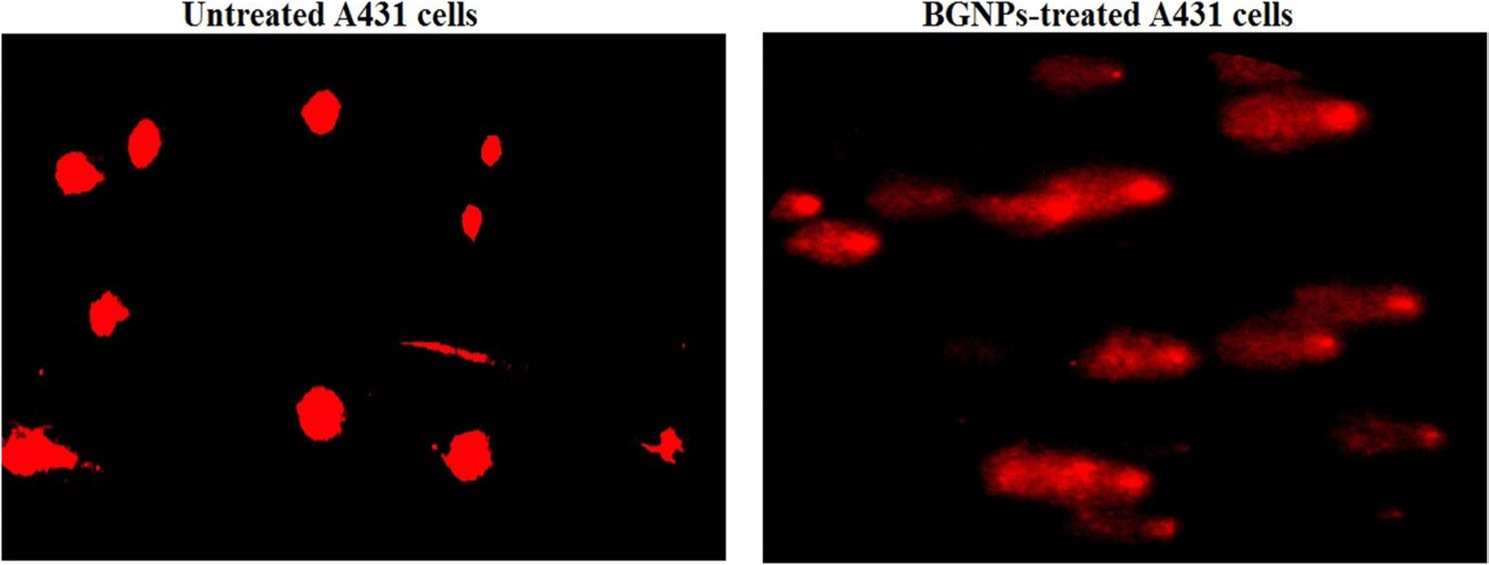
Examples for the scored Comet nuclei with intact DNA in untreated A431 cancer cells and those with damaged DNA in A431 cancer cells treated with the IC50 concentration of BGNPs (187.81 µg/ml) for 72 h. Triplicates were used. Magnification 200x

Results of the alkaline Comet assay revealed substantial genomic DNA damage in A431 epidermoid skin cancer cells following a 72-hour exposure to BGNPs at the IC50 concentration (187.81 µg/ml). This severe genotoxic effect was evidenced by highly significant increases (*p* < 0.001) in key comet assay parameters: tail length, %DNA in the tail, and tail moment, when compared to untreated control cells, as shown in Table 2 and illustrated in Fig. 3. These parameters serve as well-established indicators of DNA strand breaks and fragmentation, confirming the potent genotoxic impact of BGNPs under the experimental conditions. Microscopic examination further supported these findings: control A431 cells displayed compact nuclei with minimal DNA migration, whereas BGNPs-treated cells exhibited pronounced comet tails, hallmarks of severe DNA damage. Collectively, the quantitative data and visual comet patterns clearly demonstrate the strong DNA-damaging potential of BGNPs in A431 cancer cells (Fig. 3).

**Table 2:** Induction of DNA damage in human A431 epidermoid skin cancer cells following 72-hour exposure to the IC50 concentration (187.81 µg/ ml) of BGNPs

	Treatment (Concentration)	DNA damage indicating markers		
		Tail length (px)	%DNA in tail	Tail moment
A431 cancer cells	Untreated (0.00 µg/ml)	5.55 ± 0.95	23.67 ± 3.34	1.45 ± 0.24
	BGNPs-treated (187.81 µg/ml)	18.89 ± 0.25 ***	44.83 ± 1.63 ***	8.31 ± 0.49 ***

• Triplicates were used and results are expressed as mean ± SD

• ***: Indicates statistical significant difference from the compared untreated A431 control cells at *p* < 0.001, using *independent student t-test*

### BGNPs cause excessive ROS generation in A431 cancer cells

**Fig. 4:**
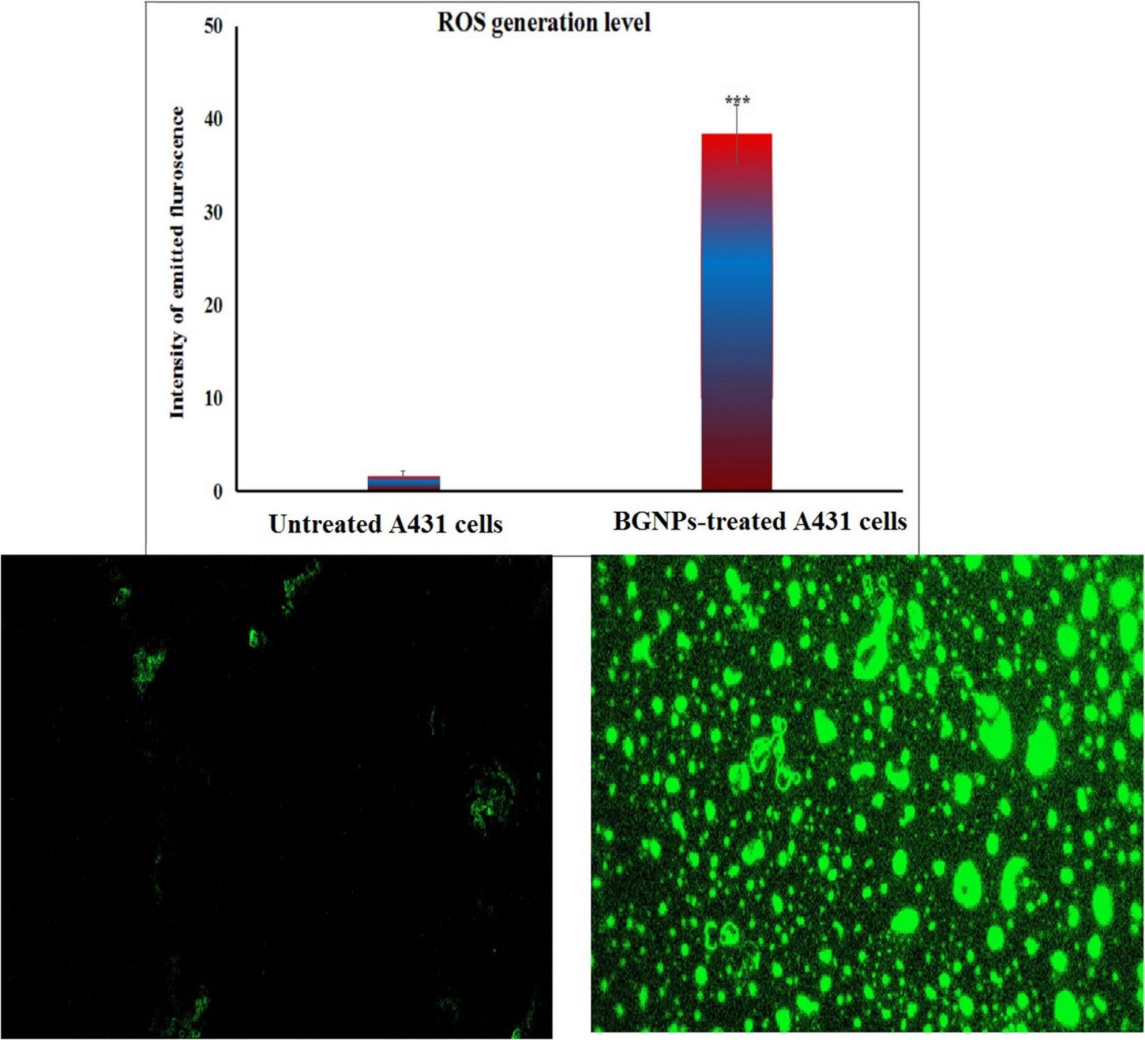
Qualitative and quantitative analysis of intracellular ROS in A431 epidermoid carcinoma cells using DCFH-DA staining and visualized by fluorescence microscopy at 200× magnification. Cells were treated with the IC50 concentration of BGNPs (187.81 µg/mL) for 72 h and compared to untreated control cells. Quantitative fluorescence intensity data are presented as mean ± SD (*n* = 3)

As depicted in Fig. 4, treatment of A431 epidermoid skin cancer cells with BGNPs at the IC50 concentration (187.81 µg/ml) for 72 h led to a pronounced increase in intracellular ROS level. This marked elevation in ROS generation was quantitatively confirmed by a highly significant rise (*p* < 0.001) in the fluorescence intensity of 2’,7’-DCFH-DA, a ROS-sensitive fluorescent dye that emits fluorescence upon oxidation by ROS. Compared to untreated control cells, BGNPs-treated A431 skin cancer cells exhibited markedly higher fluorescence signals, indicating extensive ROS accumulation within the cytoplasm (Fig. 4). These findings strongly suggest that BGNPs induce oxidative stress in A431 cancer cells, and that ROS generation is a key contributor to their cytotoxic mechanism in epidermoid skin carcinoma.

### BGNPs cause pronounced mitochondrial deplorization in A431 cancer cells

**Fig. 5:**
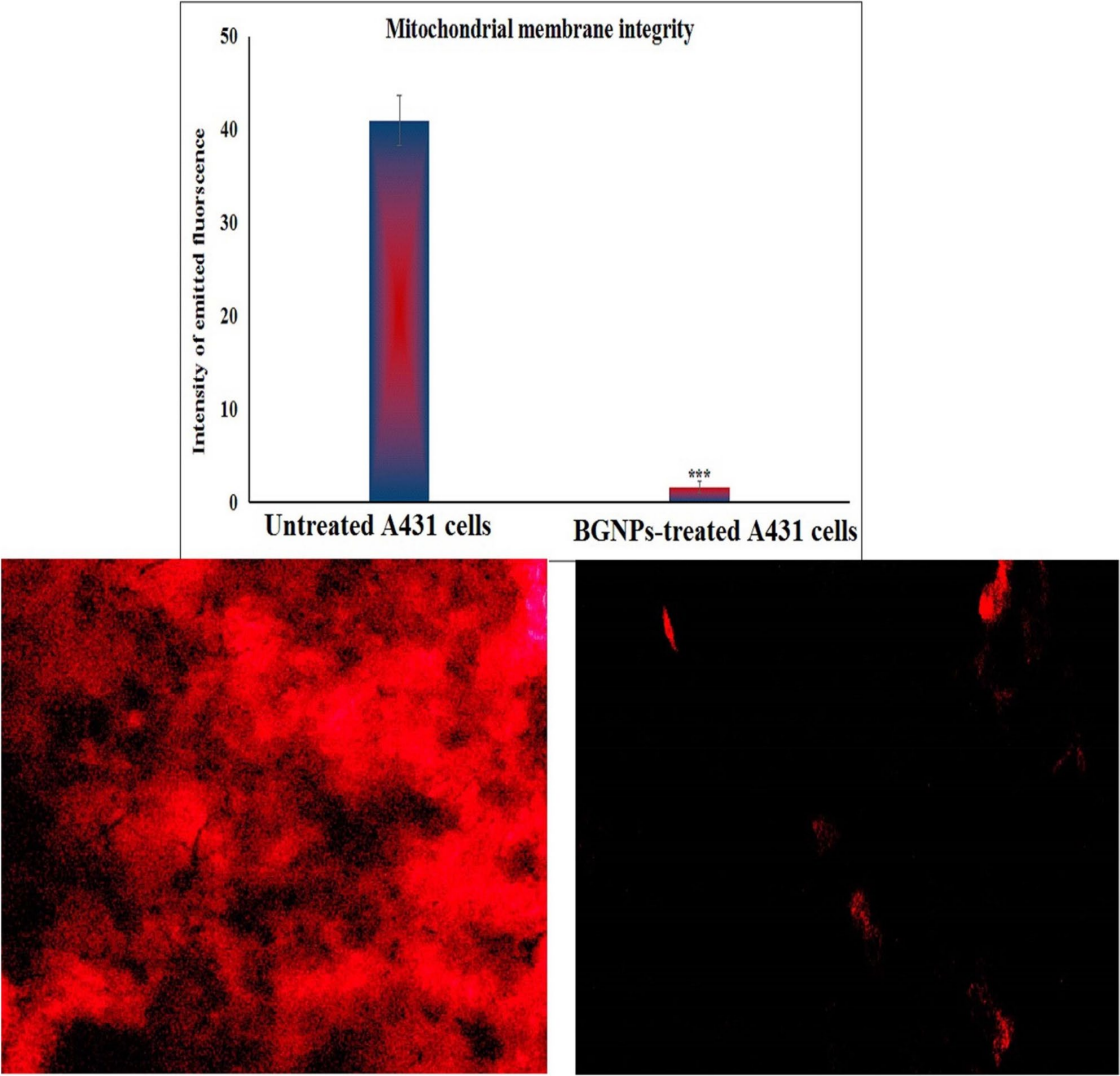
Qualitative and quantitative analysis of mitochondrial membrane potential in A431 epidermoid carcinoma cells using Rhodamine-123 staining and visualized by fluorescence microscopy at 200× magnification. Cells were treated with the IC50 concentration of BGNPs (187.81 µg/mL) for 72 h and compared to untreated control cells. Quantitative fluorescence data are presented as mean ± SD (*n* = 3)

Screening mitochondrial membrane integrity using Rhodamine-123, a fluorescent cationic dye selective for active mitochondria with intact membrane, revealed a pronounced loss of mitochondrial membrane potential in A431 skin cancer cells after 72 h of exposure to BGNPs at the IC50 concentration (187.81 µg/ml). As illustrated in Fig. 5, this significant disruption was evidenced by a statistically significant decrease (*p* < 0.001) in Rhodamine-123 fluorescence intensity in BGNPs-treated A431 cancer cells compared to untreated control cells. The diminished fluorescence reflects mitochondrial membrane depolarization, indicating substantial impairment of mitochondrial function and integrity induced by BGNPs treatment in A431 epidermoid carcinoma cells (Fig. 5).

### BGNPs stimulate apoptotic death in A431 cancer cells

**Fig. 6:**
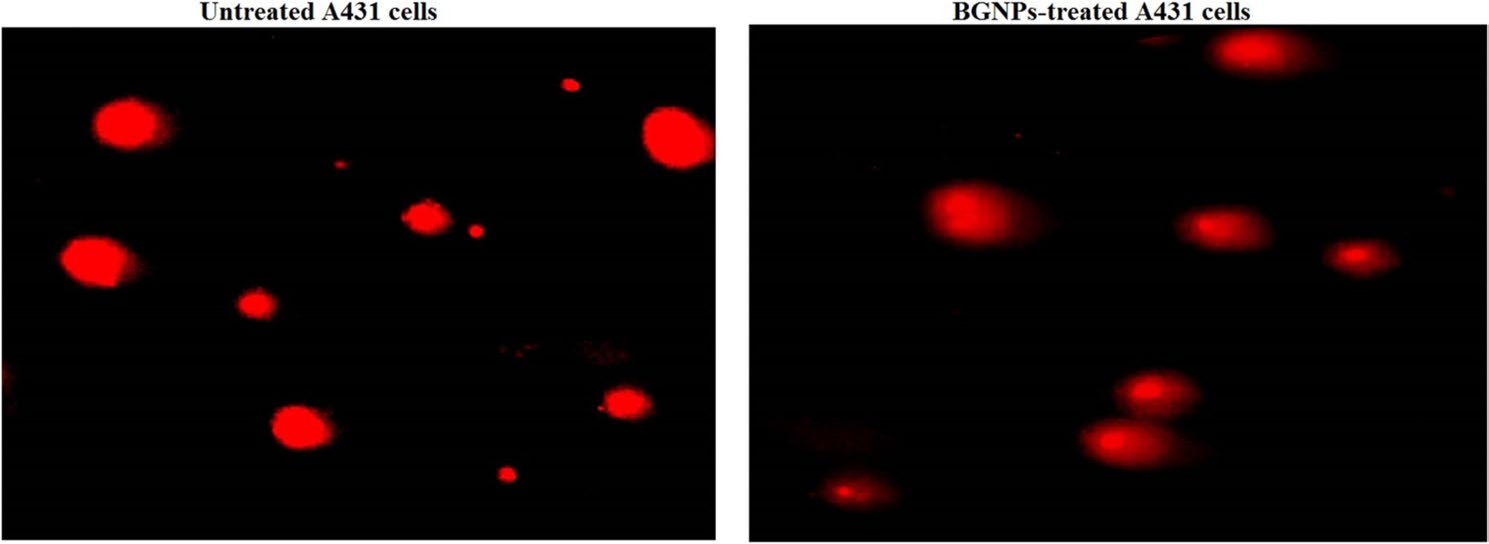
Discrimination between normal cells with intact DNA from apoptotic cells exhibiting diffused DNA using Chromatin diffusion assay in untreated and BGNPs-treated A431 epidermoid skin cancer cells following 72-hour exposure to the IC50 concentration (187.81 µg/ml). Magnification: 200×. Quantitative fluorescence data are presented as mean ± SD (*n* = 3)

**Fig. 7:**
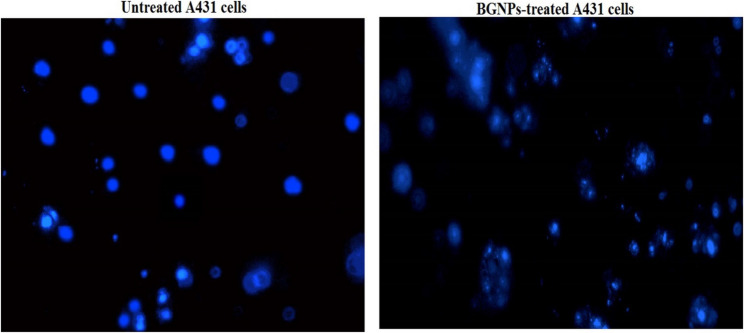
DAPI staining illustrating intact, uniformly stained nuclei in untreated A431 cancer cells and condensed or fragmented nuclei in apoptotic A431 cancer cells treated with the IC50 concentration (187.81 µg/ml) of BGNPs for 72 h. Magnification: 200×. Quantitative fluorescence data are presented as mean ± SD (*n* = 3)

Apoptosis induction in BGNPs-treated A431 skin cancer cells was confirmed through both the chromatin diffusion assay and DAPI nuclear staining, following 72-hour exposure to the IC50 concentration (187.81 µg/ml) of BGNPs. These assays collectively revealed significant apoptotic activity characterized by nuclear morphological changes and chromatin fragmentation as shown in Figs. 6 and 7. In the chromatin diffusion assay, apoptotic A431 cells were identified by the presence of characteristic chromatin diffusion halos, indicating the dispersion of fragmented DNA into the cytoplasm (Table 3). Microscopic analysis revealed that BGNPs-treated A431 cells exhibited prominent chromatin halos surrounding condensed nuclei, in sharp contrast to the compact, intact nuclei observed in untreated control cells (Fig. 6). Quantitative evaluation demonstrated highly significant increases (*p* < 0.001) in both the number of cells displaying diffusion halos and the overall percentage of apoptotic cells following BGNPs exposure as seen in Table 3. 

**Table 3:** Apoptotic incidence determined by chromatin diffusion assay in human A431 epidermoid skin cancer cells following 72-hour exposure to the IC50 concentration (187.81 µg/ml) of BGNPs

	Treatment (Concentration)	Cells with Intact DNA	Cells with Diffused DNA	%Apoptotic cells
A431 cancer cells	Untreated (0.00 µg/ml)	926.67 ± 7.37	73.33 ± 7.37	7.33 ± 0.73
	BGNPs-treated (187.81 µg/ml)	486.67 ± 35.12 ^***^	513.33 ± 35.12 ^***^	51.33 ± 3.51 ^***^

• Triplicates were used and results are expressed as mean ± SD

• ***: Indicates statistical significant difference from the compared untreated A431 control cells at < 0.001, using *independent student t-test*

DAPI staining further substantiated these findings by allowing detailed visualization of nuclear morphological changes associated with apoptosis. In untreated control cells, the nuclei appeared uniformly stained, round, and smooth, reflecting normal cellular integrity (Fig. 7). In contrast, BGNPs-treated A431 cancer cells exhibited classic apoptotic features, including chromatin condensation, nuclear shrinkage, fragmentation, and the presence of apoptotic bodies, visible as bright, condensed, and fragmented nuclei under fluorescence microscopy as depicted in Fig. 7. Quantitative analysis confirmed a statistically significant increase (*p* < 0.001) in the proportion of apoptotic nuclei in BGNPs-treated A431’cells compared to control cells (Table 4). These results conclusively demonstrate that BGNPs induce pronounced apoptosis in A431 epidermoid carcinoma cells, as evidenced by chromatin dispersion and nuclear disintegration. Such findings underscore the pro-apoptotic potential of BGNPs as a therapeutic candidate in the treatment of epidermoid skin cancer.

**Table 4:** Apoptotic incidence determined by DAPI staining assay in human A431 epidermoid skin cancer cells following 72-hour exposure to the IC50 concentration (187.81 µg/ml) of BGNPs

	Treatment (Concentration)	Intact cells	Apoptotic cells	%Apoptotic cells
A431 cancer cells	Untreated (0.00 µg/ml)	952.33 ± 8.08	47.67 ± 8.08	4.77 ± 0.81
	BGNPs-treated (187.81 µg/ml)	534.33 ± 31.37 ^***^	465.67 ± 31.37^***^	46.57 ± 3.14^***^

• Triplicates were used and results are expressed as mean ± SD

• ***: Indicates statistical significant difference from the compared untreated A431 control cells at < 0.001, using *independent student t-test*

### BGNPs significantly dysregulation the *p53*,* ND3* and *Bcl2* gene expression in A431 cancer cells

Quantitative RT-PCR analysis revealed significant dysregulation in the expression of key genes regulating apoptosis and mitochondrial function in A431 epidermoid skin cancer cells following 72-hour exposure to BGNPs at the IC50 concentration (187.81 µg/ml), as detailed in Table 5. Notably, the expression of the pro-apoptotic gene *p53* was significantly downregulated (*p* < 0.001) in BGNPs-treated A431 cancer cells compared to untreated control cells, suggesting suppression of *p53-*mediated apoptotic pathways. Conversely, both the mitochondrial gene *ND3* and the anti-apoptotic *Bcl2 gene* were markedly upregulated (*p* < 0.001) following A431 cell treatment with BGNPs. Elevated *ND3* and *Bcl2* gene expression may reflect compensatory mitochondrial responses or altered respiratory activity and mitochondrial membrane deplorization. Collectively, these remarkable alterations in *p53*,* Bcl2* and *ND3* gene expression changes suggest that BGNPs modulate apoptosis in A431 cells primarily through mitochondrial disruption and transcriptional reprogramming of apoptotic regulators. Elevated *ND3* expression may signal mitochondrial stress or compensatory shifts in respiratory activity under BGNPs-induced damage. Meanwhile, the upregulation of *Bcl2*, typically associated with cell survival, may paradoxically facilitate apoptosis when mitochondrial dysfunction and oxidative stress override its protective role. Together, these gene expression changes indicate that BGNPs impair mitochondrial homeostasis and shift the cellular balance toward mitochondrial-mediated apoptosis in A431 epidermoid carcinoma cells.

**Table 5:** Expression level of p53, ND3 and Bcl2 genes in in human A431 epidermoid skin cancer cells following 72-hour exposure to the IC50 concentration (187.81µg/ml) of BGNPs

	Treatment (Concentration)	Fold change in the expression of		
		*p53* gene	*ND3* gene	*Bcl2* gene
A431 cancer cells	Untreated (0.00 µg/ml)	1.00 ± 0.00	1.00 ± 0.00	1.00 ± 0.00
	BGNPs-treated (187.81 µg/ml)	0.48 ± 0.04 ***	1.59 ± 0.08 ***	3.06 ± 0.04 ***

• Triplicates were used and results are expressed as mean ± SD

• ***: Indicates statistical significant difference from the compared untreated A431 control cells at < 0.001, using *independent student t-test*

## Discussion

Epidermoid carcinoma of the skin poses a considerable clinical challenge due to its aggressive nature, high invasiveness, and frequent resistance to conventional treatment modalities [5]. Consequently, there is an urgent need to develop more targeted and less toxic therapeutic approaches to improve patient outcomes. Among emerging strategies, nanotechnology-based therapeutics have garnered increasing interest for their potential to selectively eliminate tumor cells while minimizing systemic side effects [31]. Among these, BGNPs have gained increasing attention due to their amorphous structure and unique physicochemical properties, including high biocompatibility, intrinsic bioactivity, and the capacity to release biologically active ions such as calcium, phosphate, and silicon. These ions are known to modulate key cellular processes, including oxidative stress responses, mitochondrial function, and cell signaling pathways. Such characteristics have underpinned their established applications in bone regeneration and antimicrobial therapies [11, 12] and provide a mechanistic basis for investigating their potential anticancer effects. Despite these advantages, the anticancer potential of BGNPs remains inadequately explored, particularly in the context of epidermoid skin cancers. Therefore, this study investigated the cytotoxic effects of BGNPs on A431 epidermoid carcinoma cells, and further examined their molecular impact on A431 cells, with emphasis on genomic DNA integrity, mitochondrial dysfunction and activation of intrinsic apoptotic pathways.

The findings of MTT assay demonstrated that BGNPs exert a potent, concentration-dependent cytotoxic effect on A431 epidermoid carcinoma cells, with an IC50 value of 187.81 µg/ml. This strong anticancer activity is consistent with our recent reports demonstrating that BGNPs can selectively inhibit the proliferation of Hepatocellular Carcinoma (Hep-G2) and non-small cell lung cancer (A549) cells while exhibiting minimal cytotoxicity toward normal, healthy cells [16–27]. Notably, these recent studies have confirmed the safety of BGNPs at comparable concentrations, showing minimal cytotoxicity toward normal human skin fibroblasts (HSF) and human melanocytes (HFB4). Such selective cytotoxicity is particularly valuable for therapeutic applications, as it reduces the risk of off-target toxicity, a major limitation associated with conventional chemotherapeutic agents [32].

The underlying mechanism of BGNPs-induced cytotoxicity in A431 epidermoid carcinoma cells was further explored by assessing genomic integrity, oxidative stress, mitochondrial function, and apoptotic markers following a 72-hour exposure to the IC50 concentration of BGNPs. The results of alkaline comet assay revealed pronounced (*p* < 0.001) DNA strand breaks and genomic instability in BGNPs-treated A431 cancer cells, indicating significant genotoxic stress. These findings are consistent with previously reported ROS-mediated DNA damage induced by various nanoparticles [33–36]. The genotoxicity observed is likely attributable to oxidative stress triggered by BGNPs, as their nanoscale dimensions and silicate-rich composition are known to facilitate intracellular reactive oxygen species (ROS) generation. This oxidative stress was evidenced by a marked increase (*p* < 0.001) in fluorescence intensity in 2’,7’-DCFH-DA–stained A431 cells treated with BGNPs, relative to untreated control cells. Given that A431 cancer cells, like many malignancies, exhibit elevated metabolic activity and inherently weakened antioxidant defense mechanisms, the excessive ROS generated by BGNPs exposure likely overwhelmed their redox buffering capacity. This redox imbalance renders cancer cells particularly susceptible to oxidative damage. Accumulated oxidative DNA lesions are well-established activators of the intrinsic (mitochondrial) apoptotic pathway, linking ROS-induced genotoxicity to programmed cell death [37, 38].

Mitochondrial dysfunction emerged as a central event in BGNPs-induced cytotoxicity. A pronounced loss (*p* < 0.001) of mitochondrial membrane potential was observed in Rhodamine-123-stained A431 cancer cells following BGNPs exposure, indicating mitochondrial depolarization and the collapse of mitochondrial integrity. This event represents a critical trigger of the intrinsic apoptotic pathway, as the loss of mitochondrial membrane potential facilitates the release of cytochrome c and subsequent activation of caspase cascades [39, 40]. These findings align with prior research demonstrating that bioactive glasses can disrupt mitochondrial function and promote apoptosis in cancer cells through ROS-mediated mechanisms and mitochondrial destabilization [41, 42]. Given the metabolic vulnerabilities of cancer cells, particularly their reliance on mitochondrial homeostasis, the ability of BGNPs to selectively impair mitochondrial integrity offers a promising therapeutic avenue for targeting treatment-resistant malignancies.

The induction of apoptosis by BGNPs in A431 cancer cells was confirmed through nuclear morphological analyses using chromatin diffusion assay and DAPI staining. BGNPs-treated A431 cancer cells displayed classical apoptotic features, including chromatin condensation, nuclear shrinkage, and fragmentation, which are well-recognized hallmarks of programmed cell death [40]. These nuclear alterations provide strong evidence for activation of the intrinsic mitochondrial apoptotic pathway, typically triggered by mitochondrial membrane disruption and exacerbated by intracellular oxidative stress [39, 43]. Collectively, our findings indicate that BGNPs induce apoptosis through a multi-step mechanism involving ROS-mediated DNA damage, mitochondrial membrane depolarization, and transcriptional dysregulation of key apoptotic and mitochondrial genes, ultimately culminating in the execution of programmed cell death. The use of chromatin-based assays provides direct visual confirmation of apoptosis, complementing other mechanistic endpoints and offering a reliable alternative to Annexin V/PI-based detection methods for morphological and mechanistic studies.

Gene expression analysis provided further insights into the molecular mechanisms underlying BGNPs-induced cytotoxicity in A431 epidermoid carcinoma cells. Notably, a significant downregulation (*p* < 0.001) of *p53* expression was detected 72 h after exposure to BGNPs at the IC50 concentration (187.81 µg/ml). This observation diverges from the classical paradigm in which *p53* serves as a central mediator of apoptosis in response to DNA damage by regulating cell cycle arrest and apoptotic gene activation [44]. However, this deviation is not entirely unexpected, as numerous cancers, including epidermoid carcinomas, frequently harbor mutated or dysfunctional *p53* pathways, which which disrupt the classical apoptotic response pathway [45, 46]. Thus, the observed BGNPs-induced apoptosis in A431 cancer cells may occur via *p53*-independent mechanisms, consistent with growing evidence that alternative apoptotic pathways can be activated in tumors lacking functional p53 [47–51].

In parallel, a marked upregulation (*p* < 0.001) of the *ND3* gene was observed in BGNPs-treated A431 cancer cells. *ND3* gene encodes a subunit of mitochondrial Complex I and plays a critical role in the electron transport chain. Its increased expression may represent a compensatory response to mitochondrial respiratory dysfunction, potentially aiming to restore electron flow under stress conditions. However, in the context of elevated ROS level, this overexpression may become maladaptive, promoting excess electron leakage and further amplifying oxidative stress and mitochondrial instability [52]. Consequently, *ND3* upregulation may intensify apoptotic signaling cascades, reinforcing the pro-apoptotic impact of BGNPs in A431 cancer cells.

Paradoxically, *Bcl-2* expression was significantly (*p* < 0.001) upregulated in BGNPs-treated A431 cancer cells. While *Bcl-2* is classically recognized as an anti-apoptotic gene that stabilizes the mitochondrial membrane and inhibits cytochrome c release, its function is highly context-dependent. In cells subjected to severe mitochondrial dysfunction and oxidative stress, as observed in BGNPs-treated cells, *Bcl-2* may lose its protective capacity or even adopt pro-oxidant properties, thereby contributing to apoptosis rather than suppressing it [39, 43, 51, 53–55]. Despite this modest upregulation, apoptosis proceeded effectively, as the cells exhibited pronounced ROS generation and mitochondrial depolarization, which are potent pro-apoptotic triggers capable of overriding *Bcl-2*-mediated survival signals. This indicates that the observed *Bcl-2* increase likely reflects a transient compensatory response rather than a mechanism of resistance. Similar patterns have been reported in other nanoparticle-based studies, where elevated *Bcl-2* levels were insufficient to prevent apoptosis under conditions of severe oxidative stress and mitochondrial disruption [51, 55]. Collectively, these findings demonstrate that BGNPs induce apoptosis through a robust, multi-step intrinsic pathway, ensuring effective cell death even in the presence of anti-apoptotic signals, and further support the potential of BGNPs as selective anticancer agents.

Moreover, the simultaneous marked elevation (*p* < 0.001) of *ND3* and *Bcl2* under BGNPs-induced stress may signify a tipping point where mitochondrial compensatory mechanisms fail, favoring apoptosis rather than survival. Collectively, these findings suggest that BGNPs promote mitochondrial-mediated apoptosis in A431 epidermoid carcinoma cells by inducing oxidative stress, disrupting mitochondrial membrane potential, and altering the expression of key mitochondrial and apoptotic regulators. The upregulation of *ND3* and *Bcl2*, in this context, likely represents a maladaptive cellular response to mitochondrial dysfunction, reinforcing the pro-apoptotic effects of BGNPs. These results expand the current understanding of BGNPs beyond their established roles in bone regeneration and antimicrobial activity, highlighting their potential as selective anticancer agents in epithelial malignancies.

The physicochemical characteristics of BGNPs play a decisive role in determining their cellular interactions and anticancer efficacy. In the current study, comprehensive characterization using XRD, DLS, and TEM revealed that BGNPs possess a nanoscale size, amorphous structure, and a negatively charged surface (− 19.89 mV), collectively providing moderate colloidal stability and facilitating efficient cellular uptake. These features are highly relevant to their biological activity: the large surface area and negative zeta potential, combined with the controlled release of biologically active ions such as calcium, silicon, and phosphate, promote the generation of ROS, disrupt mitochondrial membrane potential, and trigger apoptotic pathways in A431 epidermoid carcinoma cells [56–58]. The amorphous nature of BGNPs, in particular, enhances ion dissolution and reactivity compared to crystalline forms, further amplifying these intracellular effects [59]. Taken together, these observations indicate that the cytotoxic and mechanistic outcomes observed in A431 cells are intrinsically linked to the nanoparticles’ structural and surface characteristics, establishing a clear mechanistic connection between BGNPs design and their anticancer potential.

Taken together, this study provides novel mechanistic evidence that BGNPs induce pronounced apoptosis in A431 epidermoid skin cancer cells, primarily through mitochondrial dysfunction and ROS-mediated genomic instability. These findings support recent findings on the therapeutic potential of inorganic nanoparticles as multifunctional anticancer agents, capable of targeting multiple pathways to overcome limitations associated with conventional chemotherapy, such as drug resistance and off-target toxicity [11, 12, 51, 55]. However, it is important to acknowledge that the current study is limited to in vitro experiments and focuses on a single skin cancer cell line, which may restrict the broader applicability and generalizability of the results. To fully validate the anticancer potential of BGNPs, future studies should explore their in vivo efficacy and safety in appropriate preclinical skin cancer models. Investigating potential synergistic interactions with *Bcl−2* inhibitors and other established conventional chemotherapeutics could provide insights into combination therapy strategies. Furthermore, additional mechanistic studies are warranted to explore the complex interplay between mitochondrial signaling and apoptotic regulation, particularly in *p53*-deficient tumor contexts, which will be critical for optimizing the precision, selectivity, and therapeutic effectiveness of BGNP-based anticancer approaches.

## Conclusion

Collectively, the findings of this study provide compelling evidence that BGNPs exhibit strong, concentration-dependent cytotoxicity against A431 epidermoid skin cancer cells. This effect is mediated through a multifaceted mechanism involving ROS-driven genomic instability, mitochondrial dysfunction, and significant dysregulation of key apoptotic and mitochondrial genes, ultimately triggering intrinsic apoptosis in A431 cancer cells. At the molecular level, the response was characterized by *p53* downregulation, coupled with *ND3* and *Bcl2* upregulation, alongside chromatin condensation and nuclear fragmentation, suggesting activation of a mitochondrial-mediated, p53-independent apoptotic pathway. These findings expand the functional applications of BGNPs beyond their traditional roles in bone regeneration and antimicrobial therapy, highlighting their potential as novel, potent nanotherapeutics for epithelial malignancies. However, it is important to acknowledge that this study is limited to in vitro experiments using a single epidermoid carcinoma cell line, which may restrict the generalizability of the findings. To further substantiate these results, future studies should include in vivo validation, long-term toxicity assessments, and evaluation of BGNPs in combination with conventional chemotherapeutics. Additionally, more detailed mechanistic investigations, particularly in *p53-*deficient cancer models, will be critical to optimize BGNPs-based strategies for targeted skin cancer therapy. Overall, this study establishes a previously unreported anticancer role of BGNPs in epidermoid skin cancer, providing a foundation for future translational and preclinical research.

## Data Availability

The datasets used and/or analyzed during the current study are available from the corresponding author on reasonable request.
